# CEA-4-1BBL: CEACAM5-Targeted 4-1BB Ligand Fusion Proteins for Cis Co-Stimulation with CEA-TCB

**DOI:** 10.3390/antib14040096

**Published:** 2025-11-07

**Authors:** Christina Claus, Claudia Ferrara-Koller, Johannes Sam, Sabine Lang, Rosmarie Albrecht, Regula B. Buser, Esther Bommer, Grégory La Sala, Valeria G. Nicolini, Sara Colombetti, Marina Bacac, Pablo Umaña, Christian Klein

**Affiliations:** 1 Roche Innovation Center Zurich, Roche Pharma Research and Early Development (pRED), Wagistrasse 10, 8952 Schlieren, Switzerland; 2 Roche Innovation Center Basel, Roche Pharma Research and Early Development (pRED), Grenzacherstrasse 124, 4070 Basel, Switzerland; 3 Department of Biochemistry, Faculty of Chemistry and Pharmacy, Ludwig Maximilians University of Munich, 81377 Munich, Germany

**Keywords:** CEACAM5, 4-1BB, TNFRSF9, CD137, membrane-proximal, membrane-distal, epitope

## Abstract

Background/Objectives: T cell bispecific antibodies (TCBs) result in the activation of T cell receptor signaling upon binding to tumor antigens providing signal 1 to T cells. To enhance and sustain their activity, a co-stimulatory signal 2 is required. Here CEACAM5-targeted 4-1BBL antibody fusion proteins for combination with CEA-TCB (cibisatamab, RG7802) are described in an investigation of the relationship between the CEACAM5 epitope and T cell activity. Methods: CEACAM5-targeted bispecific 4-1BBL antibody fusion proteins (CEA-4-1BBLs) were generated based on different CEACAM5 antibodies and characterized in vitro in Jurkat-4-1BB reporter and PBMC cell assays. The impact of shed CEA on in vitro activity and cynomolgus cross-reactivity was studied. In vivo efficacy was assessed in human stem cell humanized NSG mice xenograft models bearing MKN-45 and HPAFII tumors. Results: MFE23-4-1BBL and Sm9b-4-1BBL showed superior functional activity in Jurkat-4-1BB reporter and primary T cell assays when combined with the CD3 antibody V9, whereas T84.66-LCHA-4-1BBL and A5B7-4-1BBL performed better when combined with CEA-TCB. In humanized NSG mice MKN-45 and HPAFII xenograft models, T84.66-LCHA-4-1BBL mediated the best anti-tumor efficacy. Conclusions: For the assessment of the combination of CEA-TCB with CEA-4-1BBL, co-stimulatory antibody fusion protein in vitro assays are not sufficient to fully capture the complex relationships affecting efficacy. Thus, screening with different cell assays and in vivo efficacy studies in combination with CEA-TCB are essential to select the best candidate. Based on the totality of data on the T84.66-LCHA-4-1BBL antibody fusion protein comprising the CEACAM5 antibody, T84.66-LCHA was selected as the optimal combination partner for CEA-TCB.

## 1. Introduction

Checkpoint-inhibitory and bispecific antibodies have shaped cancer immunotherapy strategies during the last decade [1,2,3,4]. Tumor-infiltrating T cells can either receive a “natural” activation signal 1 through a T cell receptor (TCR) due to its interaction with peptide–MHC complexes (endogenous immunity) or receive one “artificially” by a T cell bispecific antibody (TCB) simultaneously bound to a tumor cell surface antigen and the CD3e chain of the TCR complex leading to redirected T cell-mediated tumor cell killing (synthetic immunity) [2,5,6,7,8,9]. To enhance and sustain the activity of T cells, a co-stimulatory signal 2 is required, and various approaches have been described to provide this signal 2 co-stimulatory signal by agonizing CD28 or 4-1BB/CD137 with bispecific antibodies or fusion proteins [10,11,12,13,14]. In the clinic, promising data have been reported for several bispecific 4-1BB agonists, both as single agents and in combination with checkpoint inhibition [15,16,17,18,19,20], while clinical data for the combination with TCBs are eagerly awaited.

Carcinoembryonic antigen cell adhesion molecule 5 (CEACAM5), also known as CEA or CD66e, is a highly glycosylated cell surface protein anchored in the membrane via a glycosylphosphatidylinositol (GPI) anchor and normally functions as an adhesion molecule that can interact with CEACAM6 and CEACAM1 and plays a pivotal role in cancer biology [21,22]. CEACAM5 expression has been shown to be important for metastasis in colon cancer, but also for immune evasion of tumor cells [21,23]. CEACAM5 is overexpressed in various epithelial malignancies, including gastric, colorectal, pancreatic, breast and non-small cell lung cancer, making it an attractive drug target. Furthermore, in colorectal cancer, shed CEA is an FDA-approved diagnostic tumor marker [21,23].

We have previously described tumor stroma-targeted FAP-4-1BBL and tumor-targeted CD19-4-1BBL antibody fusion proteins that can bind simultaneously to fibroblast activation protein (FAP) or CD19, respectively, and the co-stimulatory receptor 4-1BB/CD137 [24,25]. These 4-1BBL antibody fusion proteins provide signal 2 to 4-1BB-expressing T cells, either in cis by binding to CD19-expressing malignant B cells in combination with the CD20-TCB glofitamab [26] or in trans by binding to FAP-expressing tumor fibroblasts in combination with the CEA-TCB cibisatamab [27,28]. Likewise, the combination of a CEACAM5-targeted CEA-4-1BBL fusion protein in combination with CEA-TCB was shown to strongly enhance T cell infiltration in the MKN-45 xenograft model in humanized NSG mice using the CD8-specific PET-Tracer 89Zr-Df-IAB22M2C [29]. While we have not seen major differences between 4-1BB co-stimulation in cis versus in trans, Otano et al. found that co-stimulation may be more potent when provided in cis as compared to in trans [30]. Another recent approach for 4-1BB co-stimulation in cis has been the generation of trispecific T cell engagers with integrated co-stimulation through 4-1BB binding moieties [31,32,33].

Here the design, optimization and characterization of novel CEACAM5-targeted CEA-4-1BBL antibody fusion proteins with the goal of providing signal 2 to T cells receiving their signal 1 from CEA-TCB in cis—e.g., independently of FAP expression on fibroblasts—is described. Several CEA-4-1BBL antibody fusion proteins directed against different domains of CEACAM5 were generated and tested for functionality in vitro and in vivo. The CEACAM5 antibodies chosen for the construction of the respective CEA-4-1BBL antibody fusion proteins recognize membrane-distal or membrane-proximal epitopes. Reporter T cell assays were applied for a first ranking of functionality. Targeting membrane-distal CEACAM5 epitopes mediated better T cell activation in reporter cells compared to targeting membrane-proximal epitopes when tested as a monotherapy. In contrast to the reporter cell line assays, two different PBMC-based in vitro activation assays in combination with different signal 1 providers were not able to provide a clear differentiation between the different CEACAM5 targeting antibodies. However, in MKN45 and HPAFII in vivo xenograft models in humanized NSG mice, the CEA-4-1BBL antibody fusion protein constructed with the CEACAM5 antibody T84.66-LCHA targeting a membrane-proximal CEACAM5 epitope showed the best tumor control overall in combination with CEA-TCB. Based on the totality of data, the T84.66-LCHA-4-1BBL antibody fusion protein was selected as the optimal co-stimulatory antibody fusion protein for combination with CEA-TCB.

Furthermore, the preclinical data presented indicate that reporter cell line assays can be limited in their translatability and that in order to select the best candidate screening with different cell assays, in vivo efficacy studies together with potential combination partners are essential.

## 2. Materials and Methods

Reagents: CEA–4-1BBL molecules, non-binding control DP47–4-1BBL, CEA-TCB (cibisatamab, RG7802) and huCEACAM5 peptides (NCBI EDL26990.1) were generated by Roche Innovation Centers, Zurich or Munich. CEA–4-1BBL and DP47-4-1BBL were assembled using the following polypeptide chains: (a) two ectodomains of 4-1BBL (UniProtKB P41273) fused to CL followed by a human IgG1 Fc-knob chain, (b) one ectodomain of 4-1BBL fused to CH1, (c) a CEACAM5 or DP47 VH-CH1 fused to a human IgG1 Fc-hole chain, and (d) a CEACAM5 or DP47 VL-CL. As previously described, correct chain association was enabled using knob-into-hole technology [34] and CH1-CL crossover with charge mutations [35,36], and Fc silencing was achieved with P329G LALA mutations [37]. Molecules were produced in HEK293-EBNA or CHO cells and purified by affinity chromatography followed by size exclusion chromatography as previously described [24].

The monomer content and integrity of the molecules were determined, respectively, by size exclusion on HPLC using a TSKgel UP-SW3000 column (Tosoh Biosciences LLC, King of Prussia, PA, USA) (<95%) and by CE-SDS (LabChip GXII Touch HT, Revvity, Zurich, Switzerland) (<95%). The mass of the purified molecules was confirmed by LC-MS analysis in a non-reduced state, and endotoxin content was determined. All constructs fulfilled the internal standards and were released for use in vivo. CEACAM5 Fabs were prepared by plasmin (Roche, Mannheim, Germany 10602361001) digestion of the respective human IgG1s as recently described [24]. The NABA constructs were used as antigens for the CEACAM5 Fabs. Since the CEACAM5 binders recognize different CEACAM5 domains, the following NABA constructs, containing N, A or B domains of human CEACAM1 and CEACAM5, were prepared. The constructs were purified by standard chromatographic methods (chelating chromatography and preparative SEC).

Surface plasmon resonance: All SPR experiments were performed on a Biacore T200 (Cytiva, Freiburg, Germany) at 25 °C with HBS-EP as a running buffer (0.01 M HEPES (pH 7.4), 0.15 M NaCl, 3 mM EDTA, 0.005% surfactant P20; Cytiva, Freiburg, Germany). His-specific antibody (Qiagen, Hilden, Germany, 34660) was coupled on a CM3 chip at pH 5.0 using the standard amine coupling kit (Cytiva, Freiburg, Germany). The NABA constructs were captured with a flow rate of 10 μL/minute for 30 or 40 s on flow cell 2 (Supplementary Table S1). Dilution series of CEACAM5 Fabs were passed on both flow cells at 30 μL/minute for 150 s to record the association phase. The dissociation phase was monitored for 300 s and triggered by switching from the sample solution to HBS-EP. The chip surface was regenerated after every cycle using a double injection of 60 s Glycine (pH 2.0). Bulk refractive index differences were corrected for by subtracting the response obtained on the reference flow cell, where no NABA construct was captured. The affinity constants were derived from the rate constants by fitting to a 1:1 Langmuir binding curve using the Biacore T200 Evaluation Version 3.2.2 (Cytiva, Freiburg, Germany). The results are the average of three experiments.

Cell lines: Human gastric cancer cell line MKN-45 (DSMZ ACC 409) and human colon adenocarcinoma cell lines CX-1 (DSMZ ACC 129) and HT-29 (ATCC HTB-38) were cultured in RPMI 1640 (Gibco, Thermo Fisher Scientific, Reinach, Switzerland, 42401) supplemented with 10% FBS (Gibco, Thermo Fisher Scientific, Reinach, Switzerland, 16140 or Sigma-Aldrich, Buchs, Switzerland, F4135) and 2 mM GlutaMAX (Gibco, Thermo Fisher Scientific, Reinach, Switzerland, 35050). Human pancreatic adenocarcinoma cell line HPAF-II (ATCC CRL-1997) was cultured in MEM/EBSS (Gibco, Thermo Fisher Scientific, Reinach, Switzerland, 21090) supplemented with 10% FBS, 2 mM GlutaMAX, 1% MEM-Nonessential amino acids (Sigma-Aldrich, Buchs, Switzerland, M7145) and 1 mM Sodium-Pyruvate (Sigma-Aldrich, Buchs, Switzerland, S8636). Human colorectal adenocarcinoma cell line LS-180 (ATCC CL-187) was cultured in MEM/EBSS supplemented with 10% FBS and 2 mM GlutaMAX. LoVo (ATCC CCL-229) was cultured in DMEM/F12 (Gibco, Thermo Fisher Scientific, Reinach, Switzerland, 31331) supplemented with 10% FBS and 2 mM GlutaMAX. All tumor cell lines grew adherently and were split 2-3 times per week, keeping a confluence between 20 and 80%. Jurkat-human-4-1BB-NFkB-luc2 (Promega CS196004) reporter cell line was cultured in RPMI 1640 supplemented with 10% FBS, 2 mM GlutaMAX, 1 mM Sodium-Pyruvate, 1% (*v*/*v*) MEM-nonessential Amino acid solution, 25 mM HEPES (Sigma-Aldrich, Buchs, Switzerland, H0887), 600 μg/mL G-418 (Roche, Mannheim, Germany 04727894001) and 400 μg/mL Hygromycin B (Roche, Mannheim, Germany 10843555001). The suspension cells were split twice per week, keeping a cell density of 0.2–1.2 × 106 cells/mL. CHO-k1 (ATCC CCL-61) cells were transfected with transfection plasmid pETR19312 to express cynomolgus monkey CEACAM5, and CHO-k1-cynoCEACAM5 clone 8 was selected. CHO-k1-cynoCEACAM5 clone 8 cell line was cultured in DMEM/F-12 (Gibco, Thermo Fisher Scientific, Reinach, Switzerland, 31330) supplemented with with 10% FBS and 6 μg/mL Puromycin (Gibco, Thermo Fisher Scientific, Reinach, Switzerland, A1113802) and split 3 times per week, keeping a confluence between 20 and 80%. To generate CHO-k1CEACAM5 clone 11 and CHO-k1-huCEACAM5 clone 12 cell lines CHO-k1 (ATCC CCL-61), cells were transfected with transfection plasmid pETR14582 to express human CEACAM5. Cells were cultured in DMEM/F-12 (Gibco, Thermo Fisher Scientific, Reinach, Switzerland, 31330) supplemented with 10% FBS and 500 μg/mL Zeocin (Gibco R25001) and split 3 times per week, keeping a confluence between 20 and 80%.

Cell binding assay: Tumor cell lines or CEACAM5-transfected CHO-k1 cells were harvested using 0.05% Trypsin-EDTA (Gibco, Thermo Fisher Scientific, Reinach, Switzerland, 25300) for 10 min, washed and resuspended in DPBS (Gibco, Thermo Fisher Scientific, Reinach, Switzerland, 14190). Then, 2 × 105 cells/well or 3 × 103 cells/well were seeded on a round-bottom 96-well plate (Cellstar Sigma-Aldrich, Buchs, Switzerland, 650185) or a round-bottom 384-well plate (Corning, New York, NY, USA, 3830), respectively. Cells were stained with fixable viability stain eF450 (eBioscience, Thermo Fisher Scientific, Reinach, Switzerland, 65-0863-18) according to the manufacturer’s recommendations. Cells were washed with DPBS and incubated for 1 h at 4 °C in flow cytometry staining buffer containing different concentrations of CEA-4-1BBL molecules (starting from a concentration of 300 nM, titrated in 1:5 or 1:4 dilution steps). Cells were washed with DPBS and incubated for 30 min in flow cytometry staining buffer containing 2.5 μg/mL PE-conjugated secondary antibody (Jackson ImmunoResearch, Cambridge, UK, 109-116-098). Cells were washed and fixed in PBS with 1% Paraformaldehyde for 15 min. Cells were acquired using the MACS Quant X (Miltenyi Biotec, Bergisch Gladbach, Germany) coupled to a Cytomat line 2, a 4 °C fridge and a plate handling system (Thermo Fisher Scientific, Reinach, Switzerland). Data were analyzed using FlowJo Version 10 to gate on living cells, and the median fluorescence intensity of the PE-conjugated secondary antibody was evaluated.

Reporter cell line assay: NFkB-downstream signaling after 4-1BB activation was measured by co-culturing 2 × 10^3^ Jurkat-human-4-1BB-NFkB-luc2 reporter cells with 1 × 104 tumor cells or CEACAM5-transfected CHO-k1 cells in 384-well flat-bottom white plates (Corning, New York, NY, USA 3826). Titrated CEA-4-1BBL molecules were added, and the plates were incubated for 5 h at 5% CO2 and 37 °C. For one experiment, a fixed dose of 1 nM CEA-4-1BBL was used and different concentrations of soluble CEACAM5 (BioRad, Basel, Switzerland, PHP282) were added (starting dose: 190 nM, titrated in 1:5 dilution steps). Afterwards, One-Glo Luciferase reagent (Promega, Walldorf, Germany E6110) was added, and luminescence was measured for 0.5 s/well using a Tecan Spark plate reader.

Human PBMC activation assay: Buffy coats were obtained from the Zurich blood donation center in accordance with the Declaration of Helsinki. Donors signed a written informed consent before sample collection. Peripheral blood mononuclear cells (PBMCs) were isolated by Ficoll density centrifugation as follows: buffy coat was mixed 1:1 with sterile DPBS (Gibco, Thermo Fisher Scientific, Reinach, Switzerland, 14190-136), and 35 mL of this mixture was layered over 15 mL Histopaque 1077 (Sigma-Aldrich, Buchs, Switzerland, 10771, density: 1.077 g/mL) in 50 mL Falcon containers. The containers were centrifuged for 30 min at 450× *g* at room temperature without a break. The lymphocyte fraction was harvested, pooled and washed several times with DPBS. PBMCs were frozen in 80% FBS and 10% DMSO (Sigma-Aldrich, Buchs, Switzerland, D2650) and stored in vapor phase. PBMCs were thawed, washed and directly seeded. Then, 7.5 × 10^4^ PBMCs, 2 × 10,445 Gy-irradiated MKN-45 tumor cells, 2nM agonistic human CD3 human IgG1 (clone V9) or titrated concentrations of CEA-TCB in combination with different concentrations of CEA-4-1BBL or untargeted controls were combined in 200 μL/well of assay medium in a 96-well round-bottom plate (TTP 92097). As assay medium, RPMI 1640 (GIBCO, Thermo Fisher Scientific, Reinach, Switzerland, 42401) supplemented with 10% (*v*/*v*) FBS, 2 mM GlutaMAX (Gibco, Thermo Fisher Scientific, Reinach, Switzerland, 35050), 1 mM Sodium-Pyruvat (Sigma-Aldrich, Buchs, Switzerland S8636), 1% (*v*/*v*) MEM-nonessential Amino acid solution (Sigma-Aldrich, Buchs, Switzerland, M7145) and 50 μM beta-mercaptoethanol was used. Cells were incubated under humidity at 5% CO_2_ and 37 °C. At day 4, cells were harvested, washed and stained with LIVE/DEAD Fixable Aqua Dead Cell Stain (Molecular Technology, Berlin, Germany L34957) according to the manufacturer’s recommendations. Afterwards, cells were stained in 50 μL/well flow cytometry staining buffer containing 0.3 μg/mL human CD25-APC (BioLegend, San Diego, CA, USA, 301016, clone BC96), 0.67 μg/mL human CD4-BV421 (BioLegend, San Diego, CA, USA, 300532, clone RPA-T4) and 0.67 μg/mL human CD8-APC/Cy7 (BioLegend, San Diego, CA, USA, 301016, clone RPA-T8) for 30 min at 4 °C. Cells were washed, fixed in PBS with 1% paraformaldehyde for 15 min and acquired using the Canto II (BD). Data were analyzed using FlowJo Version 10 to gate on living CD4 and CD8 T cells to determine the frequency of CD137+ or CD25+ cells of parental populations.

In vivo studies in humanized mice: The experimental study protocol was reviewed and approved by local government (code: P2011/128, date: 23 September 2016; code: ZH193-2014, date: 26 May 2020; code: ZH223/17 date: 14 May 2020). NOD.Cg-PrkdcscidIL2rgtm1Wjl/SzJ (NSG) was supplied by The Jackson Laboratory. Female NSG mice were bred by Charles River Laboratories (Lyon, France). After arrival, the animals were maintained for one week to get accustomed to the new environment and for observation. Continuous health monitoring was carried out on a regular basis. NSG mice were at the age of 4–5 weeks at the start of human stem cell engraftment and were maintained under specific-pathogen-free conditions with daily cycles of 12 h light/12 h darkness according to committed guidelines (GV-Solas; Felasa; TierschG). To generate human stem cell-engrafted (HSC)-NSG mice, NSG mice were administered 15 mg/kg busulfan (Busilvex, Pierre Fabre) intraperitoneally (i.p.). Twenty-four hours later, each mouse received an intravenous (i.v.) injection of 1 × 10^5^ human CD34+ cord blood cells (purchased from STEMCELL Technologies). At 15–17 weeks post-engraftment, the mice were screened and only mice with more than 25% human CD45+ cells and a T cell count greater than 80 cells/μL were enrolled in the studies. For tumor inoculation, 1 × 10^6^ tumor cells were resuspended in a 1:1 mixture of RPMI 1640 (Gibco, Thermo Fisher Scientific, Reinach, Switzerland, 42401) and GFR Matrigel (Corning, New York, NY, USA, 734-0269) to a total volume of 100 μL and injected subcutaneously into the flank. Tumor volume was calculated using the formula: V = length × width^2^ × 0.5, and it was used to randomize groups one day before treatment. Tumor-bearing mice (average tumor size: 200 mm^3^, randomized groups) were treated intravenously with vehicle or 2.5 mg/kg CEA-TCB alone or in combination with 1, 3 or 10 mg/kg CEA–4-1BBL molecules. The dose for CEA-TCB was determined in previous experiments as optimal for xenograft studies in (HSC)-NSG mice and translatable. Tumor volume was calculated using the formula: V = length × width^2^ × 0.5.

Flow cytometry measurement ex vivo: For ex vivo flow cytometry analysis, tumor single-cell suspensions were prepared using the gentleMACS Octo Dissociator (Miltenyi Biotec, Germany) according to the manufacturer’s protocol and subsequently analyzed by flow cytometry. Formaldehyde-fixed tumor tissues were utilized for immunohistochemistry. Digested tumor single-cell suspensions were stained with 1:500 diluted live/death fixable blue dye (molecular probes: L-23105) according to the manufacturer’s recommendations. Cells were washed and stained in flow cytometry staining buffer containing mouse CD45-AF700 (BioLegend, San Diego, CA, USA, 103128, clone 30-F11), human CD3-BV605 (BD 750985) and human CD4-BV421 (BioLegend, San Diego, CA, USA, 317434, clone OKT4), all diluted 1:300 and incubated for 30 min at 4 °C in the dark. Cells were washed, fixed and permeabilized using Permeablization buffer 10× from the FoxP3 Transcription Buffer staining kit (eBioscience, Thermo Fisher Scientific, Reinach, Switzerland, 00-5523-00). Cells were stained in perm-buffer containing 1:300 diluted human FoxP3-PE and incubated for 30 min at 4 °C in the dark. Cells were acquired with a 5-laser BD Fortessa flow cytometer and analyzed.

Immunohistology ex vivo: Immunohistochemical staining was performed in formalin-fixed paraffin-embedded tissue (FFPET) as described in [3,4] with human CD8 (Cell Marque Tissue Diagnostic, Sigma-Aldrich, Buchs, Switzerland, clone SP16) and human CD3 (Thermo Fisher Scientific, Reinach, Switzerland, MA5-13473, clone C8/144B). Human CD3+ and human CD8+ cells were quantified with Definiens Tissue Studio 3.0 (Definiens, Gilde Healthcare, Munich, Germany).

CEACAM5 modelling: Inter-domain centroid and center-of-mass distance calculations: In order to calculate typical distances between domains of the full-length human CEACAM5 protein, the alphafold2 model AF-P06731-F1-v6 was applied [38,39], which was aligned with available published CEACAM5 CryoEM structures of the N-terminal domain (PDB: 2QSQ, 2QST, 2VER) [40] and A3-B3 domain (PDB: 8BW0) [41]. All structures were analyzed using MOE (Molecular Operating Environment (MOE); 2024.0601 Chemical Computing Group ULC, 910-1010 Sherbrooke St. W., Montreal, QC H3A 2R7, Canada, 2025). All solved individual domains were initially superimposed based on structure and sequence similarities to evaluate the folding reliability of the AlphaFold model. Sequences corresponding to the N-term (A.A. 1–34) and C-term tail (A.A. 677–702) of the CEACAM5 AlphaFold model ranked with very low confidence (per residue confidence score pLDDT < 50, as disclosed in the model description file) were removed from the structure for further analysis. Next, structures were prepared using the protonate3D function and refined using an RMS gradient of 0.1 kcal/mol/A2. Domains were annotated and color-coded according to the figure legends. In order to measure a meaningful inter-domain distance, a centroid was defined as the geometric center corresponding to the average position of all atoms in each domain. Similarly, mass-weighted positions of all atoms in the domain were defined as centers of mass. Inter-centroid and inter-center-of-mass distances were next measured and compared to the maximal paratopic distance between the CDRL1 and CDRH2 of the solved CryoEM structure of tusamitamab bound to CEACAM5 (PDB:8BW0) [41].

Images: The pictograms shown below were designed with Biorender.com.

Statistics: Binding and activation values were baseline-corrected. Curves were analyzed by calculating the area under the curve (AUC), and AUC values were then used to calculate significant differences using an unpaired one-way ANOVA test. For in vivo efficacy studies, statistical analysis was performed using 1-way analysis of variance (ANOVA), and significant *p* values are indicated as follows: ns: not significant; * *p* < 0.05; ** *p* < 0.01; *** *p* < 0.001; **** *p* < 0.0001.

## 3. Results

### 3.1. CEA-4-1BBL Antibody Fusion Protein Design

**Figure 1:**
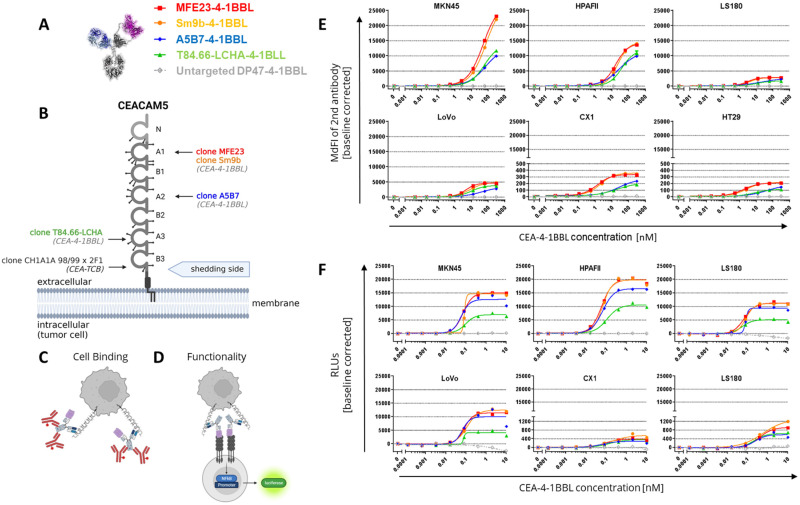
Evaluating binding to CEACAM5-expressing tumor cells and crosslinking functionality in an analogous reporter cell line assay. (**A**) Tested CEA-4-1BBL bispecific molecules. The CEACAM5 binding Fab is indicated in blue, the split trimeric 4-1BB ligand in purple and the Fc portion in gray. (**B**) Different CEACAM5 epitopes and binders. (**C**) Schematic illustration of binding assay. (**D**) Schematic illustration of functional reporter cell line assay. (**E**) CEA-4-1BBL binding to different CEACAM5-expressing tumor cells. CEA-4-1BBL molecules were incubated with the indicated tumor cell lines. Specific binding was detected using a PE-conjugated secondary antibody, and median of fluorescence intensity (MdFI) was measured by flow cytometry and baseline-corrected. Each point is the mean of technical duplicates. One of three similar experiments is shown. (**F**) CEA-4-1BBL molecules were titrated and incubated for 5 h with the reporter cell line Jurkat-human-4-1BB-NFkB-luc and different tumor cells in a 1:1 ratio. Emitting luminescence after substrate addition was measured for 0.5 s/well as relative light units (RLUs). Each point is the mean of technical duplicates. One of three independent and similar experiments is shown.

Following the recently described 4-1BBL antibody fusion protein design [24], different CEA-4-1BBL antibody fusion proteins were generated using five different human CEACAM5-specific antibodies for tumor targeting (Figure 1A). As a non-tumor-targeted control, the previously published DP47-4-1BBL fusion protein was applied [24]. This construct does not bind to the cell surface of cancer cells. CEA-4-1BBL was specifically designed for combination with CEA-TCB, which comprises the human CEA-specific antibody CH1A1A98/99x2F1 and targets the membrane-proximal CEACAM5 domain B3 [27,42]. Thus, to allow the combination of CEA-TCB with CEA-4-1BBL, only CEACAM5-specific antibodies, which do not share the same CEACAM5 epitope and do not compete for binding with CEA-TCB, were selected (Figure 1B and Figure S1). The following antibodies known from the literature were chosen: (1) the humanized antibody T84.66-LCHA binding to CEACAM5 domain A3 [43,44,45], (2) the parental antibody A5B7 and its humanized version huA5B7 binding to the membrane-proximal CEACAM5 domain A2 [46,47], and (3) the parental antibody MFE23 [48,49] and its humanized, stabilized and affinity-matured version Sm9b binding to CEACAM5 domain A1 [50]. All five of these CEACAM5 antibodies recognize soluble CEACAM5, a product induced by glycosylphosphatidylinositol-phospholipase D cleavage activity [51], as their epitopes are above the shedding site (Figure 1B). In contrast, the epitope of CH1A1A98/99x2F1 applied in CEA-TCB is close to the shedding site, and binding to soluble CEACAM5 as well as inhibition by soluble CEACAM5 is considered negligible, as it has been shown for its parental antibody, PR1A3 [52]. To confirm that simultaneous multiple Fab binding to CEACAM5 domains is possible from a structural point of view, inter-domain distances relative to the B3 domain of the AlphaFold model of human CEACAM5 were calculated (Supplementary Figure S1A,B). Measurements show that a typical Fv paratope length scores shorter than inter-domain distances (Supplementary Figure S1C), further supporting that simultaneous binding to individual domains occurs.

### 3.2. CEA-4-1BBL In Vitro Characterization

The affinity determination of the CEACAM5-targeting Fab fragments for CEACAM5 NABA constructs covering the N-A1-B2-A2 domains (Supplementary Table S1) via surface plasmon resonance (SPR) demonstrated that T84.66-LCHA and A5B7 have the highest and MFE23 and Sm9b the lowest affinities (Table 1). To test the cellular binding of the different CEA-4-1BBL antibody fusion proteins, six different human tumor cell lines were chosen to be representative for high to low CEACAM5 surface expression, as described previously [27,42]. The ability of the different CEA-4-1BBL fusion proteins to bind these CEACAM5+ human cancer cell lines was assessed by flow cytometry using a binding assay, as described in Figure 1C. The measured binding as median fluorescence intensity (MdFI) showed that Sm9b-4-1BBL and its parental variant, MFE23-4-1BBL, displayed similar MdFI values, which were higher than for A5B7-4-1BBL or T84.66-LCHA-4-1BBL (Figure 1E, Supplementary Table S2). The discrepancy between affinity determined by surface plasmon resonance and cell binding is likely caused by a different epitope accessibility on cells and/or differences in the glycosylation profiles between recombinant NABA-CEACAM5 and CEACAM5 expressed on tumor cell surfaces. To test if these differences in tumor cell binding translate into differences in functionality, the Jurkat-human-4-1BB-NFkB-luc2 reporter assay illustrated in Figure 1D was applied using the same tumor cell line panel as applied in the cell binding assay (Figure 1F, Supplementary Table S3). MFE23-4-1BBL and Sm9b-4-1BBL displayed comparable functionality similar to the cell binding results. On the other hand, A5B7-4-1BBL and T84.66-LCHA-4-1BBL, which showed lower overall cell binding, displayed lower activity in the reporter cell assay, with T84.66-LCHA-4-1BBL showing the lowest activity, while A5B7-4-1BBL performed more similarly to MFE23-4-1BBL and Sm9b-4-1BBL (Figure 1F).

**Figure 2:**
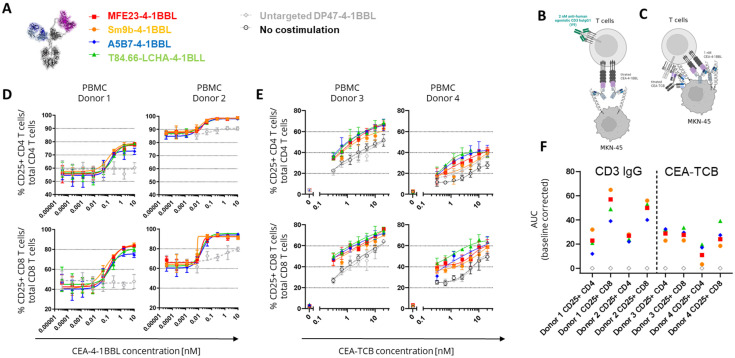
Co-stimulatory activity of CEA-4-1BBL molecules on primary human T cells. (**A**) Tested CEA-4-1BBL bispecific molecules. (**B**) Schematic display of the assay with CD3 IgG1. (**C**) Schematic display of the assay with CEA-TCB. (**D**) CEA-4-1BBL molecules were titrated and incubated with human PBMCs from two different donors, 45 Gy-irradiated MKN-45 tumor and 2 nM agonistic CD3 human IgG1 V9 antibody delivering signal 1, as indicated. After 4 days, T cells were analyzed by flow cytometry. The frequency of CD25 upregulation in the parental CD4 or CD8 T cell population was determined as indicated. Each point is the mean of technical triplicates. Error bars indicate the standard deviation. Only one experiment was performed. (**E**) Human PBMCs from two different donors were incubated with 45 Gy-irradiated MKN-45 tumor cells, titrated CEA-TCB and 1 nM CEA- 4-1BBL molecules, as indicated. After 4 days, T cells were analyzed by flow cytometry. The frequency of CD25 upregulation in the parental CD4 or CD8 T cell population was determined. Each point is the mean of technical triplicates. Error bars indicate the standard deviation. Only one experiment was performed. (**F**) Area under the curve (AUC) was calculated from both assays and baseline-corrected by subtracting the AUC value from the DP47-4-1BBL control. The mean is shown.

In subsequent experiments it was tested whether these differences in functional activity can also be observed in primary T cell assays. Human PBMCs obtained from different healthy donors were activated in the presence of CEACAM5-expressing MKN-45 tumor cells in the presence of 2 nM of the agonistic human CD3 IgG1 antibody V9 (Figure 2B) or a titration of CEA-TCB (Figure 2C). This activation step was introduced to induce sufficient 4-1BB expression, as resting T cells do not express 4-1BB constitutively [24]. The timing of this assay was optimized in pre-experiments, and four days was found to be the optimal time point. Interestingly, in both assays, in the presence of TCR signal 1, no major differences between the tested CEA-4-1BBL fusion proteins were observed, as were seen in the Jurkat-human-4-1BB-NFkB-luc2 reporter cell line assays with CEA-4-1BBL as a monotherapy (Figure 2D,E, Supplementary Tables S4 and S5). In the primary T cell assays, only minimal differences were observed. However, there was a trend suggesting that MFE23-4-1BBL and Sm9b-4-1BBL showed better performance when combined with the agonistic human CD3 antibody V9 (Figure 2D), whereas T84.66-LCHA-4-1BBL and A5B7-4-1BBL appeared to perform better when combined with CEA-TCB (Figure 2F). This demonstrates that the resolution of PBMC activation assays is smaller and different to the reporter cell assay. Furthermore, it demonstrated that the signal 1 provider, CD3 antibody versus TCB, may have an impact on the activity of the CEA-4-1BBL fusion proteins.

**Figure 3:**
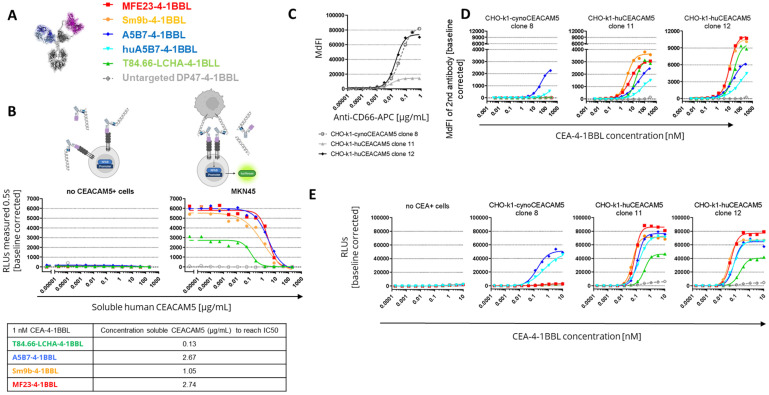
Inhibitory capacity of soluble CEACAM5 and cynomolgus monkey cross-reactivity. (**A**) Tested CEA-4-1BBL bispecific molecules. (**B**) Quantities of 1 nM of indicated CEA-4-1BBL molecules were incubated for 5 h with the reporter cell line Jurkat-human-4-1BB-NFkB-luc, with or without MKN-45 tumor cells, in a 1:1 ratio and in the presence of titrated concentrations of soluble human CEACAM5. After substrate addition, emitting luminescence was measured for 0.5 s/well as relative light units (RLUs) and baseline-corrected. Each point is the mean of technical duplicates. IC50 values were calculated and are displayed. Only one experiment was performed. (**C**) CHO-k1 cells were stably transduced with cynomolgus monkey (cynoCEACAM5) or human CEACAM5 (huCEACAM5). To compare expression levels between the different clones, a human/cynomolgus monkey cross-reactive CD66e-APC detection antibody was used for flow cytometry analysis. The concentration of the CD66e-APC detection antibody is displayed against the median fluorescence intensity (MdFI). Each point indicates the mean of technical duplicates. One of five independent and similar experiments is shown. (**D**) CEA-4-1BBL molecules were incubated with the indicated transgenic CHO-k1 cells and detected with a PE-conjugated secondary antibody. The MdFI was measured by flow cytometry, and values were baseline-corrected. Each point is the mean of technical duplicates. One of five independent and similar experiments is shown. (**E**) CEA-4-1BBL molecules were titrated and incubated for 5 h with the reporter cell line Jurkat-human-4-1BB-NFkB-luc and in the absence or presence of transgenic CHO-k1 cells in a 1:1 ratio. After addition of substrate, emitting luminescence was measured for 0.5 s/well as RLUs. Each point is the mean of technical duplicates. One of three independent and similar experiments is shown.

As the PBMC-based assays had a longer incubation time of four days than the reporter cell line assay with an incubation time of five hours, subsequent experiments investigated whether the accumulation of soluble CEACAM5 may result in a reduction in the binding to cell-bound CEACAM5 and could provide an explanation for the findings. Indeed, overexpression of CEACAM5 in colorectal cancer correlates with soluble CEACAM5 concentrations in serum, and in cancer patients concentrations from 0.002 to 5.187 ug/mL have been reported, with soluble CEACAM5 values generally correlating with advanced cancer stages [53]. To test whether soluble human CEACAM5 can lead to inhibition of the functionality of the different CEA-4-1BBL fusion proteins (Figure 3A), a fixed concentration of 1 nM CEA-4-1BBL was chosen (Figure 3B). After adding increasing concentrations of soluble human CEACAM5 in the Jurkat-human-4-1BB-NFkB-luc2 reporter cell line assay, the IC50 values were evaluated. In these experiments the strongest inhibition was seen for T84.66-LCHA-4-1BBL and the lowest for A5B7-4-1BBL and MF23-4-1BBL (Figure 3B). Therefore, the functional differences in the different assays could not be explained by the sole impact of soluble CEACAM5.

### 3.3. Cynomolgus Cross-Reactivity

To select the best molecule for further development, the cynomolgus cross-reactivity of the different CEA-4-1BBL molecules was determined. Cynomolgus cross-reactivity has already been shown for human 4-1BBL [24], so only the cynomolgus cross-reactivity of the different CEACAM5 antibodies was determined. For this purpose, CHO-k1 cell lines stably expressing either human or cynomolgus monkey CEACAM5 were generated. To compare the cynoCEACAM5 expression levels of the different chosen CHO-k1 clones, a human/cynomolgus cross-reactive CEACAM5 (CD66e) APC-conjugated detection antibody was chosen. Based on the expression data, CHO-k1-cynoCEACAM5 clone 8 with good cynoCEACAM5 expression was selected for subsequent experiments. Furthermore, CHO-k1-huCEACAM5 clone 12 and clone 11 were selected as controls (Figure 3C). With these three cell lines, cell binding assays were performed (Figure 3D, Supplementary Table S6). Only A5B7-4-1BBL showed cyno-cross-reactivity, although with reduced binding to cynoCEACAM5. A5B7-4-1BBL bound much more weakly to CHO-k1-cynoCEACAM5 clone 8 (EC50 = 41.8 nM) than to CHO-k1-huCEACAM5 clone 12 (EC50 = 21.69 nM), although both clones displayed similar CEACAM5 expression levels (Figure 3C). As A5B7 was the only cyno-cross-reactive clone, it was humanized and converted into the CEA-4-1BBL format (huA5B7-4-1BBL). The humanization led to slightly reduced binding to CHO-k1-huCEACAM5 cells; however, unfortunately, the binding to CHO-k1-cynoCEACAM5 clone 8 decreased dramatically as a result of the humanization (Figure 3D, Supplementary Table S6). To test if the low binding to cynoCECAM5 had an impact on functionality, the same transgenic CHO-k1 cell lines were used in the functional Jurkat-human-4-1BB-NFkB-luc2 reporter assay (Figure 3E, Supplementary Table S7). As expected, only A5B7-4-1BBL and huA5B7-4-1BBL induced reporter cell line activation in the presence of CHO-k1-cynoCEACAM5. However, both antibody fusion proteins showed a lower activity with CHO-k1-cynoCEACAM5 than with CHO-k1-humanCEACAM5 controls. Interestingly, compared to A5B7-4-1BBL, huA5B7-4-1BBL functionality was not reduced as much as the binding to CHO-k1-cynoCEACAM5 would have suggested. Furthermore, no significant differences between CHO-k1-huCEACAM5 clone 11 and CHO-k1-huCEACAM5 clone 12 were observed in the reporter assay (Figure 3E), although both cell lines have different CEACAM5 expression (Figure 3C). It is important to mention that the CHO-k1 cell lines express high levels of CEACAM5 compared to tumor cell lines (Figure 1D) and that saturation of the reporter cell line activity with CHO-k1-huCEACAM5 clone 11 might have been reached.

### 3.4. In Vivo Efficacy Studies

**Figure 4:**
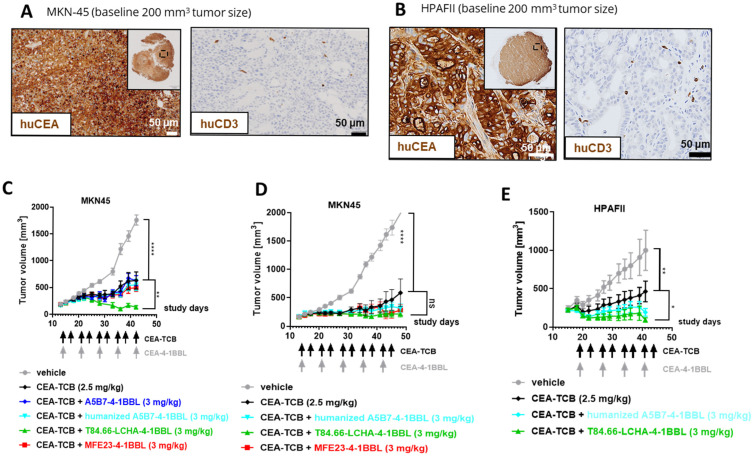
CEA-4-1BBL-mediated tumor growth inhibition in humanized mice (**A**,**B**) The human CEACAM5 expression and human CD3+ cell infiltration measured by IHC on FFPET at a tumor size of 200 mm^3^ before treatment of MKN-45 or HPAFII tumors are shown. (**C**) CD34+ human cord blood humanized NOG mice received a subcutaneous injection of MKN-45 tumor cells. When a tumor volume of around 200 mm^3^ was reached, the mice were randomized and treated with the vehicle (gray filled circle) or 2.5 mg/kg CEA-TCB (black filled diamond) twice per week. Four of the six groups received a combination treatment of 2.5 mg/kg CEA-TCB twice per week and 3 mg/kg CEA-4-1BBL once per week (the different colors indicate CEA-4-1BBL molecules implementing different CEACAM5 binders). The schedule of treatment is indicated along the x-axis; tumor growth in mm^3^ is indicated along the y-axis. Each point indicates the mean for 10 mice; error bars show the standard error of the mean (SEM). (**D**) A repetition of the experiment shown in C with indicated molecules. (**E**) A repetition of the experiment shown in C but with HPAFII tumor cells instead of MKN45 with indicated molecules. Statistical analysis was performed using 1-way analysis of variance (ANOVA), and significant *p* values are indicated as follows: ns: not significant; * *p* < 0.05; ** *p* < 0.01; **** *p* < 0.0001.

**Figure 5:**
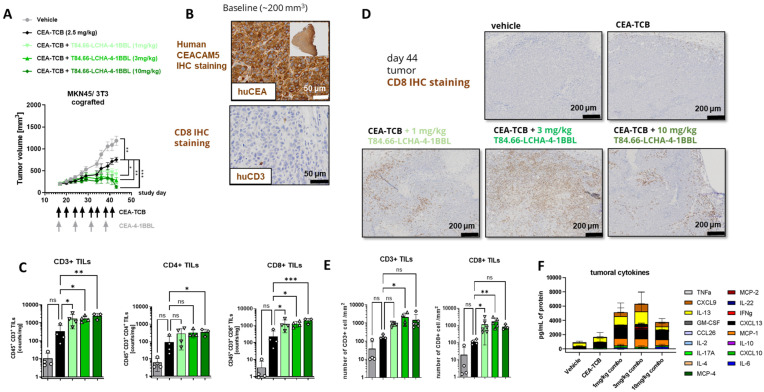
CEA-4-1BBL mediated increased CD8 T cell accumulation in a dose-dependent manner. (**A**) CD34+ human cord blood humanized NOG mice received subcutaneous injection of MKN-45 tumor cells and murine 3T3 fibroblasts. When a tumor volume of around 200 mm^3^ was reached, mice were randomized and were treated with vehicle (gray) or 2.5 mg/kg CEA-TCB (black) twice per week. Three of the five groups received a combination treatment of 2.5 mg/kg CEA-TCB twice per week and 1m 3 or 10 mg/kg CEA-4-1BBL once per week (different shades of green). The schedule of treatment is indicated along the x-axis; tumor growth in mm^3^ is indicated along the y-axis. Each point indicates the mean of 10 mice; error bars show the standard error of the mean (SEM). (**B**) The human CEACAM5 expression measured by IHC on FFPET at a tumor size of 200 mm^3^ before treatment is shown. (**C**) At the endpoint (day 44), tumors were isolated and weighted. Three to four of ten tumors were digested and analyzed by flow cytometry. The counts of CD45+ CD3+ T cells, CD45+ CD3+ CD4+ T cells and CD45+ CD3+ CD8+ T cells per mg tumor are shown. Each symbol indicates an individual mouse; the bars indicate the mean, and the error bars the standard deviation (SD). (**D**) A total of 3-6 tumors per group were harvested at the endpoint (day 44); the tissue was formalin-fixed, paraffin-embedded and stained by immunohistochemistry (IHC) for human CD8 to monitor the tumor infiltration of CD8+ T cells. Tissue sections were scanned, and whole scans were analyzed by Definiens. One representative picture per group is shown. (**E**) The CD3+ and CD8+ T cell counts per square millimeter of immunohistochemistry are shown. Each symbol represents one individual mouse; the bars indicate the mean, and the error bars indicate the standard deviation (SD). Significance was calculated using unpaired one-way ANOVA with Tukey’s multiple comparison test. ns: not significant; * = *p* ≤ 0.05; ** *p* < 0.01. (**F**) Tumor was digested and centrifuged at endpoint day 44. Human cytokines were measured from supernatant using a 24plex R&D. For vehicle, CEA-TCB and CEA-TCB + 3 mg/kg T84.66-LCHA-4-1BBL, tumors from n = 4 mice were analyzed; for CEA-TCB + 1 mg/kg T84.66-LCHA-4-1BBL, tumors from n = 5 mice were analyzed; and for CEA-TCB + 10 mg/kg T84.66-LCHA-4-1BBL, tumors from n = 3 mice were analyzed. Measured cytokines are shown in different colors as the mean ± SD per treatment group. Statistical analysis was performed using 1-way analysis of variance (ANOVA), and significant *p* values are indicated as follows: ns: not significant; * *p* < 0.05; ** *p* < 0.01; *** *p* < 0.001.

Finally, the potential of the different CEA-4-1BBL antibody fusion proteins as signal 2 providers in combination with CEA-TCB was tested in vivo in human CD34+ stem cell humanized NSG mice bearing subcutaneous human gastric adenocarcinoma MKN-45 or human pancreatic adenocarcinoma HPAFII xenografts. Both models exhibit comparable and high CEACAM5 expression and low CD3 T cell infiltration at baseline (Figure 4A,B), making them xenograft models with good translational potential for colorectal, gastric and pancreatic cancers. All treated mice exhibited reduced tumor growth relative to the vehicle. Notably, only T84.66-LCHA-4-1BBL consistently demonstrated significant tumor growth control across the performed experiments as a combination partner with CEA-TCB when compared with CEA-TCB monotherapy (Figure 4C–E). To establish this activity further and evaluate the dose–response relationship, T84.66-LCHA-4-1BBL was evaluated at three different doses in the MKN45/3T3 co-grafting tumor model in humanized NSG mice [24]. Administration of T84.66-LCHA-4-1BBL at all three tested doses (1, 3 and 10 mg/kg) resulted in the enhancement of CEA-TCB-mediated tumor growth inhibition (Figure 5A), exhibiting a clear dose-dependent trend. Furthermore, treatment with all three doses resulted in comparable immuno-pharmacodynamic effects in the tumor, as evidenced by flow cytometric analysis of human CD4^+^ and CD8^+^ T cell infiltration (Figure 5C). Consistently, immunohistochemistry (Figure 5D,E) demonstrated a significant increase in intratumoral human T cell density. Lastly, an increased accumulation of cytokines within the tumor was observed in the combination groups over CEA-TCB monotherapy at the study endpoint on day 44 (Figure 5F).

## 4. Discussion

CEACAM5 is a well-studied marker for colorectal cancer, and soluble CEA serves as an FDA-approved diagnostic tumor marker [22,23]. Due to its broad and relatively tumor-selective expression, CEACAM5 has a high potential to serve as a target for different antibody-based cancer (immuno-) therapies. Several CEA-targeted TCBs have reached clinical trials, including CEA-TCB (cibisatamab) [27] and MEDI-565/AMG211 [54]. Clinical data have been reported for CEA-TCB as a monotherapy and in combination with the PD-L1 antibody atezolizumab [55]. Recently, promising data were reported for the TopoI payload-based CEACAM5-specific antibody drug conjugate precemtabart tocentecan in a Phase 1 study in colorectal cancer patients [56]. Notably, CEACAM5 has been described as displaying immune cell-inhibitory functions. For example, CEACAM5 expressed by tumor cells can interact with CEACAM1 expressed by NK cells and lead to the inhibition of NK cell-mediated tumor target cell killing [57]. Furthermore, it has been proposed that CEACAM5 can directly interact with CD1d via its B3 domain and with CD8α via its N-domain, leading to the formation of suppressive CD8 regulatory T cells [58]. CEACAM5 expression in pancreatic cancer also correlates with impairment of the tumoricidal function of M1 macrophages and neutrophils, although the mechanism is not fully understood [59].

Here different CEACAM5-specific antibodies directed against different epitopes/domains were tested to see which of them when used as targeting antibodies in CEA-4-1BBL fusion proteins would lead to the best CEA-4-1BBL-mediated tumor control in combination with CEA-TCB. Interestingly, in vitro MFE23-4-1BBL and Sm9b-4-1BBL (targeting membrane-distal CEACAM5 epitopes) performed superiorly in binding to tumor cells and in the functional Jurkat-human-4-1BB-NFkB-luc2 reporter cell assay. Similarly, when combined with a CD3 antibody as a signal 1 provider, both molecules had a tendency to perform better in the PBMC activation assay. In contrast, T84.66-LCHA-4-1BBL targeting a membrane-proximal epitope showed weaker binding on tumor cells and the lowest activity in the reporter cell assay. However, when combined with CEA-TCB as a signal 1 provider, T84.66-LCHA-4-1BBL mediated the best activity overall, both in a human PBMC assay and in vivo in two different xenograft models in human CD34+ stem cell humanized NSG mice.

The location of the tumor-target epitope, whether the binder-specific epitope is proximal or distal to the membrane, can influence T cell bispecific antibody function. Membrane-proximal epitopes are generally considered beneficial for TCBs, as they favor the formation of functional synapses between T and tumor cells. Indeed, Bluemel and colleagues used a MCSP-TCB comparing different MCSP-specific binders which recognized membrane-proximal, central and membrane-distal domains [60]. Membrane-proximal binding led to the strongest T cell-mediated tumor cell killing. Similarly, an EpCAM-TCB was tested, where not the EpCAM antibody but the location of the recognized epitope was modified by introducing spacers to mimic a more distal location of the same EpCAM epitope. In this example, creating a membrane-distal location led to the loss of bispecific-mediated T cell cytotoxicity [60]. Li and colleagues have studied FcRH5-TCB. The membrane-proximal-domain FcRH5 binder demonstrated the strongest in vitro functionality; however, a membrane-distal binder mediated similar tumor cell killing when the epitope was moved to a membrane-proximal position through genetic engineering [61]. Finally, Chen and colleagues investigated BCMA and Flt3 TCBs and their specific lysis capacity depending on the epitope localization, affinity and molecular design of the bispecific antibodies [62]. They showed that molecular design and tumor-target epitope location can decouple cytotoxicity and cytokine release and must be coordinated to achieve the best synapse and tumor killing results [62]. Antigen-binding affinities, on the other hand, appear to be positively correlated with cytotoxicity and cytokine release [62]. It is therefore reasonable to speculate that T84.66-LCHA-4-1BBL, by virtue of binding to the membrane-proximal A3 domain, is the optimal combination partner for CEA-TCB, recognizing the membrane-proximal B3 domain and thus enabling optimal synapse formation (Figure 1B and Figure S1).

In contrast, the improved performance of membrane-distal CEACAM5-binding CEA-4-1BBL bispecific molecules in vitro in the Jurkat-human-4-1BB-NFkB-luc2 reporter cell line assay was surprising, as in general a smaller interspace that better mimics natural synapse formation is considered superior. In order to trigger TCR signaling in Jurkat reporter cells, physical immobilization and crosslinking appear to be more important than the actual synapse formation. Accordingly, the ranking observed in the Jurkat-human-4-1BB-NFkB-luc2 reporter cell line assay was not reflected in the PBMC-based activation assays. In vivo, T84.66-LCHA-4-1BBL displayed the best tumor growth control in combination with CEA-TCB over several in vivo studies and models. Importantly, testing CEA-4-1BBL activity in the in vitro PMBC activation assay or in vivo requires the presence of an active signal 1, whereas the Jurkat reporter cell line works without additional signal 1 and therefore ultimately may not reflect the impact of functional synapse formation in the presence of bispecific molecules. Therefore, the reporter cell line assay in this particular case cannot be considered the assay of choice for the selection of bispecific lead candidates, and broader testing, including TCBs as combination partners and in vivo experiments, is recommended. Based on the totality of the PBMC activation and in vivo data in combination with CEA-TCB, T84.66-LCHA-4-1BBL was chosen as the lead. Due to the discontinuation of the clinical development of CEA-TCB (cibisatamab, RG7802), further preclinical development of T84.66-LCHA-4-1BBL was halted; however, these findings may be of relevance to the development of future CEACAM5-targeted co-stimulatory agents and their combination with T cell engagers.

## Figures and Tables

**Table 1:** Affinity constants of CEACAM5 Fabs for human CEACAM5 NABA constructs. The results are the average of three experiments.

Anti-CEACAM5 Fab	Human CEACAM5 NABA Construct	ka (1/Ms)	kd (1/s)	K_D_ (M)	SD
MFE23	huCEACAM1(A2,B1)-huCEACAM5(N, A1) avi His	9.2 × 10^4^	1.1 × 10^−3^	1.2 × 10^−8^	1.3 × 10^−9^
Sm9b	huCEACAM1(A2,B1)-huCEACAM5(N, A1) avi His	8.1 × 10^4^	9.0 × 10^−4^	1.1 × 10^−8^	1.3 × 10^−9^
A5B7	huCEACAM1(N,A2)-huCEACAM5(A2,B2) avi His	3.6 × 10^5^	3.1 × 10^−4^	8.6 × 10^−10^	5 × 10^−11^
huA5B7	huCEACAM1(N,A2)-huCEACAM5(A2,B2) avi His	1.9 × 10^5^	4.6 × 10^−4^	2.5 × 10^−9^	5.5 × 10^−11^
T84.66-LCHA	huCEACAM1(N,A2)-huCEACAM5(A3,B3) avi His	8.9 × 10^5^	1.1 × 10^−4^	1.2 × 10^−10^	3.0 × 10^−11^
CH1A1A 98/99 × 2F1	huCEACAM1(N,A2)-huCEACAM5(A3,B3) avi His	9.0 × 10^5^	8.1 × 10^−3^	9.1 × 10^−9^	7.9 × 10^−10^

## Data Availability

Data are available upon reasonable request from the authors.

## Supplementary Materials

The following supporting information can be downloaded at https://www.mdpi.com/article/10.3390/antib14040096/s1: Table S1: NABA constructs and conditions used for determining the affinity constants of the CEACAM5 Fabs via surface plasmon resonance (SPR); Table S2: EC50 in nM and area under the curve (AUC) values of binding curves for different CEACAM5-expressing tumor cell lines shown in Figure 1E; Table S3: EC50 in nM and area under the curve (AUC) values of activation curves for the Jurkat-human-4-1BB-NFkB-luc reporter cell line in the presence of different CEACAM5-expressing tumor cell lines shown in Figure 1F; Table S4: EC50 in nM and area under the curve (AUC) values of activation curves for the activation assay for PBMCs from two different donors shown in Figure 2D; Table S5: Area under the curve (AUC) values of activation curves for the activation assay for PBMCs from two different donors shown in Figure 2E; Table S6: EC50 in nM and area under the curve (AUC) values of binding curves for different transgenic cynomolgus monkey or human CEACAM5-expressing CHO-k1 cell lines shown in Figure 3C and 3D; Table S7: EC50 in nM and area under the curve (AUC) values of binding curves for different transgenic cynomolgus monkey or human CEACAM5-expressing CHO-k1 cell lines shown in Figure 3E; Figure S1: Inter-domain distance of human CEACAM5 AlphaFold model. Centroids, defined as geometric centers of individual domains, were calculated and depicted as red dots within each individual domain and used for distance calculation relative to the B3 centroids (depicted as green lines in A and reported in B). Similarly, centers of mass accounting for each domain’s atom mass were calculated and used for relative distance measurement (reported in B). Structural overlay of B3-A3 domains with solved CryoEM structure of tusamitamab bound to CEAMCAM5 (gray, PDB: 8BW0) allowed the calculation of maximal FV paratope distance between the CDRL1 and CDRH2 of a typical Fv antibody (C).

## Abbreviations

The following abbreviations are used in this manuscript:

CEA	Carcinoembryonic Antigen 5
CEACAM5	Carcinoembryonic Antigen Cell Adhesion Molecule 5
FAP	Fibroblast Activation Protein
MdFI	Median Fluorescence Intensity
PBMC	Peripheral Blood Mononuclear Cell
PET	Positron Emission Tomography
SPR	Surface Plasmon Resonance
TCB	T Cell Bispecific Antibody
TCR	T Cell Receptor
