# Supplementary material for: CEA-4-1BBL: CEACAM5-Targeted 4-1BB Ligand Fusion Proteins for Cis Co-Stimulation with CEA-TCB
Source: Antibodies (Basel). 2025 Nov 7;14(4):96. doi: 10.3390/antib14040096 (PMC12641798; doi:10.3390/antib14040096)
Supplement: Supplementary file 1 [file antibodies-14-00096-s001.zip › antibodies-3926378-supplementary.pdf]

Figure S1

A

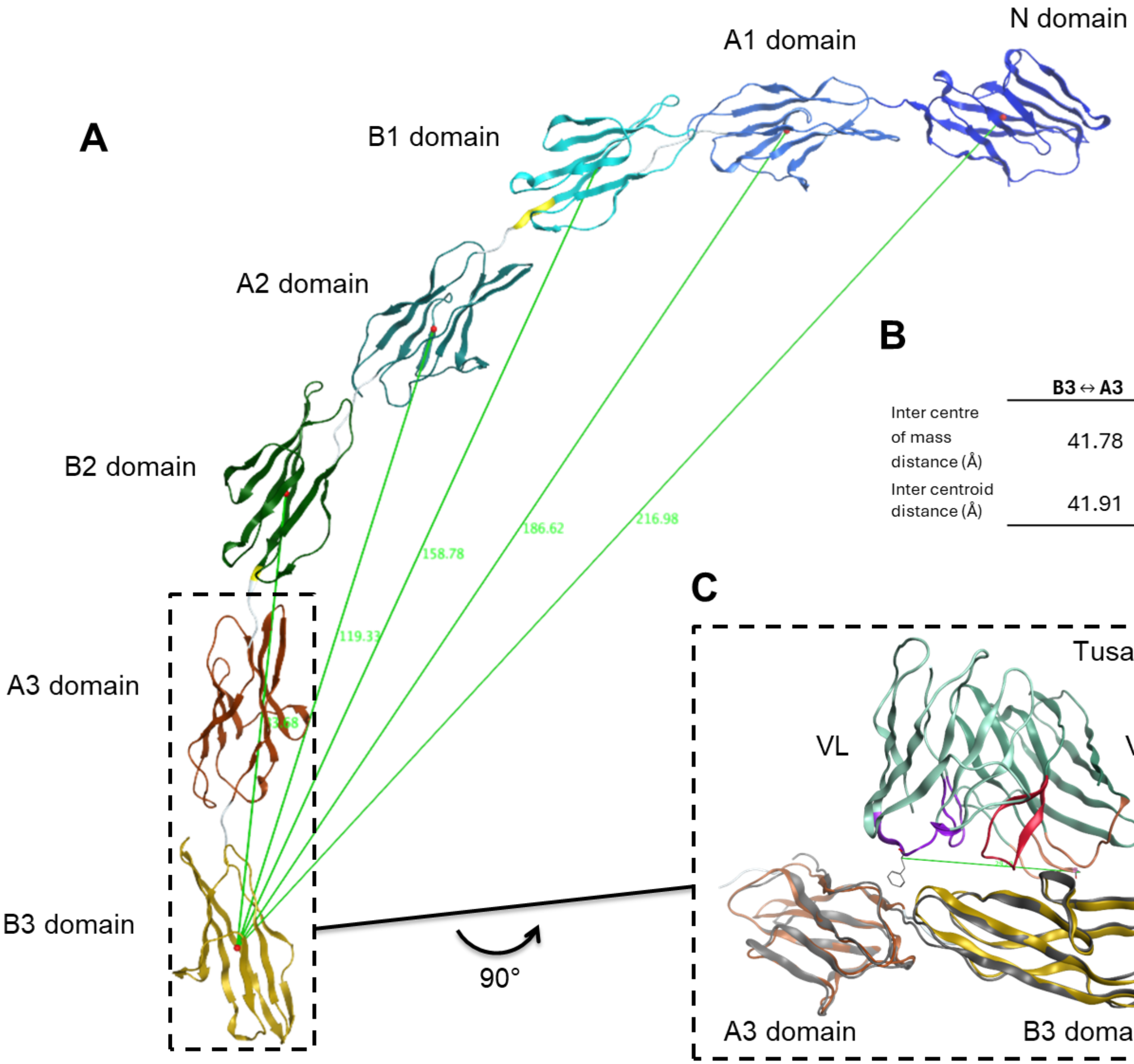

B

	B3 ↔ A3	B3 ↔ B2	B3 ↔ A2	B3 ↔ B1	B3 ↔ A1	B3 ↔ N
Inter centre of mass distance (Å)	41.78	83.72	119.17	158.79	186.61	216.93
Inter centroid distance (Å)	41.91	83.68	119.33	158.78	186.62	216.98

C

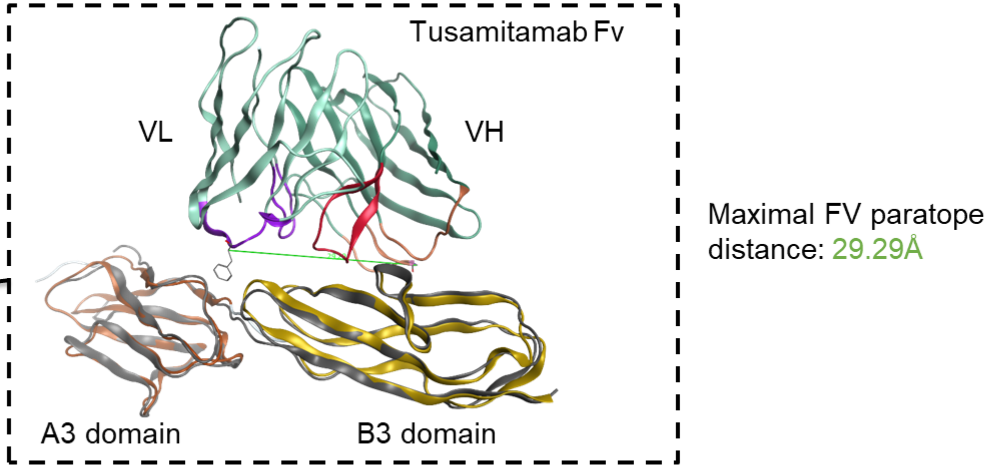

Figure S1: Inter-domain distance of human CEACAM5 AlphaFold model. Centroids, defined as geometric centers of individual domains, were calculated and depicted as red dots within each individual domain and used for distance calculation relative to the B3 centroids (depicted as green lines in A and reported in B). Similarly, centers of mass accounting for each domain's atom mass were calculated and used for relative distance measurement (reported in B). Structural overlay of B3-A3 domains with solved CryoEM structure of tusamitamab bound to CEAMCAM5 (gray, PDB: 8BW0) allowed the calculation of maximal FV paratope distance between the CDRL1 and CDRH2 of a typical Fv antibody (C).

Table S1: NABA constructs and conditions used for determining the affinity constants of the anti-CEACAM5 Fabs via Surface plasmon resonance (SPR). The conditions were optimized for every anti-CEACAM5 Fab.

**Anti-CEACAM5**	**NABA construct used for SPR**<br>**Orange = human CEACAM5 (huCEACAM5)**<br>**Blue = human CEACAM1 (huCEACAM1)**		**SPR conditions used**
**MFE23-Fab**	huCEACAM1(A2,B1)-huCEACAM5(N, A1) avi His	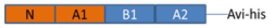	40 seconds capture time<br>800 - 6.25 nM Fab
**Sm9b-Fab**	huCEACAM1(A2,B1)-huCEACAM5(N, A1) avi His	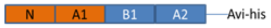	40 seconds capture time<br>800 - 6.25 nM Fab
**A5B7-Fab**	huCEACAM1(N,A2)-huCEACAM5(A2,B2) avi His	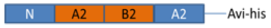	30 seconds capture time<br>400 - 3.13 nM Fab
**huA5B7-Fab**	huCEACAM1(N,A2)-huCEACAM5(A2,B2) avi His	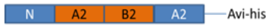	30 seconds capture time<br>400 - 3.13 nM Fab
**T84.66-LCHA-Fab**	huCEACAM1(N,A2)-huCEACAM5(A3,B3) avi His	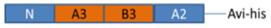	30 seconds capture time<br>100 - 0.78 nM Fab
**CH1A1A 98/99 x 2F1-Fab**	huCEACAM1(N,A2)-huCEACAM5(A3,B3) avi His	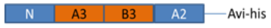	30 seconds capture time<br>200 - 1.56 nM Fab

Table S2: EC50 in nM and area under the curve (AUC) values of binding curves to different CEACAM5 expressing tumor cell lines shown in Figure 1E. Shown is the mean of technical duplets of one experiment.

**Binding**	**Tumor cell line**	**MFE23-4-1BBL**	**Sm9b-4-1BBL**	**A5B7-4-1BBL**	**T84.66-LCHA-4-1BBL**	**DP47-4-1BBL**
EC50 [nM]	MKN45	49.2	75.1	67.7	61.6	n.d.
	HPAFII	16.5	22.5	27.3	39.4	n.d.
	LS180	3.0	3.9	21.4	10.5	n.d.
	LoVo	4.5	8.1	42.1	9.5	n.d.
	CX-1	1.3	1.9	26.7	19.1	n.d.
	HT29	1.7	2.5	48.4	8.0	n.d.
AUC	MKN45	24462	21184	10314	11311	10
	HPAFII	18743	17347	12777	11304	11
	LS180	5977	5458	3401	2632	26
	LoVo	8779	8209	3390	6036	12
	CX-1	870	863	374	328	41
	HT29	512	491	194	196	12

Table S3: EC50 in nM and area under the curve (AUC) values of activation curves of Jurkat-human-4-1BB-NFκB-luc reporter cell line in the present of different CEACAM5 expressing tumor cell lines shown in Figure 1F. Shown is the mean of technical duplets of one experiment.

Reporter cell line activation	Tumor cell line	MFE23-4-1BBL	Sm9b-4-1BBL	A5B7-4-1BBL	T84.66-LCHA-4-1BBL	DP47-4-1BBL
EC50 [nM]	MKN45	0.071	0.084	0.058	0.094	n.d.
	HPAFII	0.059	0.066	0.066	0.109	n.d.
	LS180	0.069	0.082	0.082	0.055	n.d.
	LoVo	0.082	0.110	0.065	0.076	n.d.
	CX-1	0.086	0.188	0.060	0.139	n.d.
	HT29	0.396	0.571	0.231	0.215	n.d.
AUC	MKN45	32101	30165	29146	14133	209
	HPAFII	44968	44373	36305	20936	2294
	LS180	24576	23804	20148	12212	2905
	LoVo	24236	24630	23837	10196	4219
	CX-1	838	1014	653	705	136
	HT29	1301	1606	1074	1054	216

Table S4: EC50 in nM and area under the curve (AUC) values of activation curves of the PBMC activation assay from two different donors shown in Figure 2D. Shown is the mean of technical triplicates of one experiment.

PBMC activation assay	Read out	MFE23-4-1BBL	Sm9b-4-1BBL	A5B7-4-1BBL	T84.66-LCHA-4-1BBL	DP47-4-1BBL
EC50 [nM]	Donor 1 %CD25+ CD4	0.156	0.114	0.113	0.229	n.d.
	Donor 1 %CD25+ CD8	0.136	0.077	0.105	0.197	n.d.
	Donor 2 %CD25+ CD4	0.024	0.017	0.014	0.020	n.d.
	Donor 2 %CD25+ CD8	0.016	0.008	0.017	0.016	n.d.
AUC	Donor 1 %CD25+ CD4	341	349	330	338	318
	Donor 1 %CD25+ CD8	312	320	295	304	256
	Donor 2 %CD25+ CD4	506	507	501	504	479
	Donor 2 %CD25+ CD8	430	436	420	433	380

Table S5: Area under the curve (AUC) values of activation curves of the PBMC activation assay from two different donors shown in Figure 2E. Shown is the mean of technical triplicates of one experiment.

**Tumor cell line**	**MFE23-4-1BBL**	**Sm9b-4-1BBL**	**A5B7-4-1BBL**	**T84.66-LCHA-4-1BBL**	**DP47-4-1BBL**	**No costimulator**
Donor 3 %CD25+ CD4	96	90	99	99	67	71
Donor 4 %CD25+ CD8	55	47	61	63	44	32
Donor 3 %CD25+ CD4	109	104	111	115	81	85
Donor 4 %CD25+ CD8	86	80	89	101	61	62

Table S6: EC50 in nM and area under the curve (AUC) values of binding curves to different transgenic cynomolgus monkey or human CEACAM5 expressing CHO-k1 cell lines shown in Figure 3C and 3D. Shown is the mean of technical duplets of one experiment.

Binding	Tumor cell line	Anti-CD66-APC	MFE23-4-1BBL	Sm9b-4-1BBL	A5B7-4-1BBL	huA5B7-4-1BBL	T84.66-LCHA-4-1BBL
EC50 [nM]	CHO-k1-cynoCEACAM5 clone 8	0.039	n.d.	n.d.	41.9	>800	n.d.
	CHO-k1-huCEACAM5 clone 11	0.010	6.0	3.1	31.1	61.9	16.3
	CHO-k1-huCEACAM5 clone 12	0.019	11.2	10.4	21.7	~150	25.0
AUC	CHO-k1-cynoCEACAM5 clone 8	112 770	7	35	2 113	296	30
	CHO-k1-huCEACAM5 clone 11	28 963	5 356	7 282	2 989	1 513	4 034
	CHO-k1-huCEACAM5 clone 12	125 483	16 412	15 866	7 821	4 371	10 852

Table S7: EC50 in nM and area under the curve (AUC) values of binding curves to different transgenic cynomolgus monkey or human CEACAM5 expressing CHO-k1 cell lines shown in Figure 3E. Shown is the mean of technical duplets of one experiment.

Reporter cell line activation	Tumor cell line	MFE23-4-1BBL	Sm9b-4-1BBL	A5B7-4-1BBL	huA5B7-4-1BBL	T84.66-LCHA-4-1BBL
EC50 [nM]	CHO-k1-cynoCEACAM5 clone 8	n.d.	n.d.	0.17	0.53	n.d.
	CHO-k1-huCEACAM5 clone 11	0.08	0.05	0.12	0.16	0.37
	CHO-k1-huCEACAM5 clone 12	0.05	0.03	0.08	0.09	0.22
AUC	No CEACAM5 expressing cells	2 730	2 812	2 089	2 596	2 826
	CHO-k1-cynoCEACAM5 clone 8	3 947	3 926	89 755	63 359	3 516
	CHO-k1-huCEACAM5 clone 11	181 931	161 824	146 145	131 372	67 077
	CHO-k1-huCEACAM5 clone 12	177 352	164 619	135 302	137 491	67 724
